# Rapid and dynamic subcellular reorganization following mechanical stimulation of *Arabidopsis *epidermal cells mimics responses to fungal and oomycete attack

**DOI:** 10.1186/1471-2229-8-63

**Published:** 2008-06-02

**Authors:** Adrienne R Hardham, Daigo Takemoto, Rosemary G White

**Affiliations:** 1 Plant Cell Biology Group, Research School of Biological Sciences, The Australian National University, Canberra, ACT 2601, Australia; 2 Plant Pathology Laboratory, Graduate School of Bioagricultural Sciences, Nagoya University, Chikusa, Nagoya, 464-8601, Japan; 3 Division of Plant Industry, C.S.I.R.O., Canberra, ACT 2601, Australia

## Abstract

**Background:**

Plant cells respond to the presence of potential fungal or oomycete pathogens by mounting a basal defence response that involves aggregation of cytoplasm, reorganization of cytoskeletal, endomembrane and other cell components and development of cell wall appositions beneath the infection site. This response is induced by non-adapted, avirulent and virulent pathogens alike, and in the majority of cases achieves penetration resistance against the microorganism on the plant surface. To explore the nature of signals that trigger this subcellular response and to determine the timing of its induction, we have monitored the reorganization of GFP-tagged actin, microtubules, endoplasmic reticulum (ER) and peroxisomes in *Arabidopsis *plants – after touching the epidermal surface with a microneedle.

**Results:**

Within 3 to 5 minutes of touching the surface of *Arabidopsis *cotyledon epidermal cells with fine glass or tungsten needles, actin microfilaments, ER and peroxisomes began to accumulate beneath the point of contact with the needle. Formation of a dense patch of actin was followed by focusing of actin cables on the site of contact. Touching the cell surface induced localized depolymerization of microtubules to form a microtubule-depleted zone surrounding a dense patch of GFP-tubulin beneath the needle tip. The concentration of actin, GFP-tubulin, ER and peroxisomes remained focused on the contact site as the needle moved across the cell surface and quickly dispersed when the needle was removed.

**Conclusion:**

Our results show that plant cells can detect the gentle pressure of a microneedle on the epidermal cell surface and respond by reorganizing subcellular components in a manner similar to that induced during attack by potential fungal or oomycete pathogens. The results of our study indicate that during plant-pathogen interactions, the basal defence response may be induced by the plant's perception of the physical force exerted by the pathogen as it attempts to invade the epidermal cell surface.

## Background

Early studies of plant-pathogen interactions documented an increase in the activity of cytoplasmic streaming and accumulation of cytoplasm beneath the invading pathogen cell as the first structural manifestation of the response of plants to microorganisms on their surface [1-4]. Cytoplasmic aggregation is accompanied by reorganization of cytoskeletal and endomembrane components which become focused on the infection site [5-8]. This cytoplasmic reorganization is followed by thickening and strengthening of the cell wall to form wall appositions, or papillae, beneath the invading pathogen [9]. Wall appositions develop as the result of localized deposition of callose and site-directed secretion of other cell wall components and anti-microbial compounds including phenolics, silicon, H_2_O_2 _and pathogenesis-related proteins [10-12]. Once formed, wall appositions constitute an enhanced physical and chemical barrier against invading pathogens and are a central component of the basal defence response [9,13,14]. This basal defence response is non-specific in that it is mounted at the onset of non-host, incompatible or compatible interactions. The penetration resistance that is achieved successfully thwarts attack by most eukaryotic microorganisms.

Localized secretion of wall materials and toxins depends on the actin cytoskeleton which is believed to be responsible for the distribution of ER and for transport of dictyosomes, secretory vesicles and other cell components to the infection site [15-19]. The dynamics of cytoskeleton and endomembrane reorganization during pathogen attack have been explored in living cells through studies of GFP-tagged cell components [20-24]. These studies indicate that, having been initiated, rearrangement of plant cell components can occur rapidly. For example, extensive changes in the distribution and morphology of the ER have been followed during a 15-minute period from the onset of reorganization beneath a *Phytophthora sojae *hypha growing across the outer epidermal cell wall [20]. It is, however, difficult to determine the timing of induction of subcellular response in relation to the stage of infection.

In light of the lack of specificity in induction of subcellular reorganization following inoculation with non-adapted, avirulent and virulent fungi or oomycetes, we hypothesized that the response could be triggered by physical detection of pressure exerted by the pathogen as it attempts to penetrate the plant epidermis. In the study reported here, we have tested this hypothesis by monitoring the response of GFP-tagged components in epidermal cells of *Arabidopsis thaliana *cotyledons when they are touched gently with a fine microneedle. Our results show that within 3 to 5 minutes, plant cells respond to this mechanical stimulation, displaying similar subcellular reorganization to that observed during pathogen invasion.

## Results

### Image collection

The organization of GFP-tagged cell components was imaged by collecting z-series of optical sections through the cortical cytoplasm underlying the outer epidermal cell wall before and after touching the cotyledon surface with a microneedle. The depth of the cortical cytoplasm and the number of optical sections required to scan through it were determined before bringing the needle into contact with the outer surface of the cell wall. In preliminary trials, it was found that z-series comprised of 5–7 sections were optimal as this meant that optical sections were taken at intervals of 0.8–1.0 μm and the time between z-series was only 20–40 seconds.

**Figure 1:**
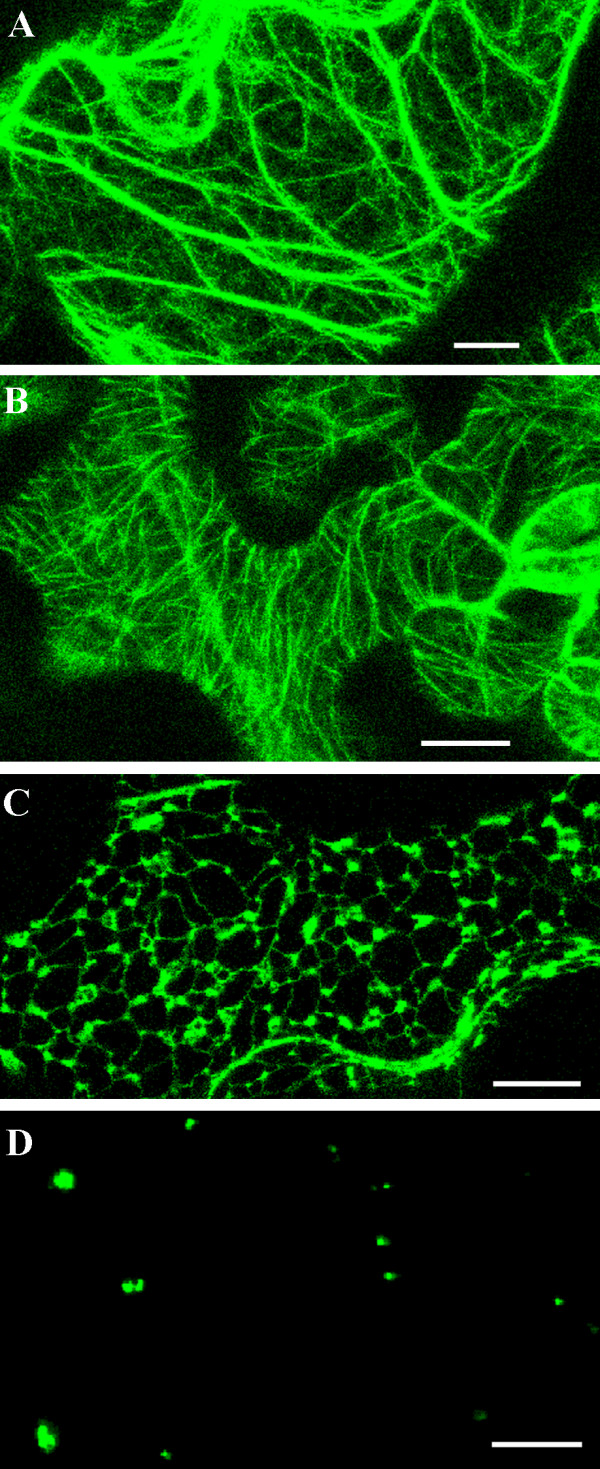
**The organization of GFP-tagged cell components in untreated cells**. The distribution of actin (A), microtubules (B), endoplasmic reticulum (ER) (C) and peroxisomes (D) in *Arabidopsis thaliana *expressing hTalin-GFP [20], TUA6-GFP [70], KKXX-GFP [71] and PTS-GFP [46], respectively. Images are maximum projections of z-series optical sections taken through the cortical cytoplasm underlying the outer wall of cotyledon epidermal cells before being touched with the microneedle. Bars = 10 μm.

After imaging the arrangement of GFP-tagged components in the epidermal cell before needle contact (Fig. 1), the needle was brought into contact with the cotyledon surface and z-series were collected continuously for the next 20–60 minutes. Image collection much beyond about an hour was hampered by quenching of GFP fluorescence. The needle was judged to have made contact with the outer surface of the cell wall by comparing the focal planes of the needle tip and the fluorescent organelles within the cortical cytoplasm and by noting a slight change in the focus of the wall and underlying cytoplasm as the needle was slowly advanced. If the needle were advanced too far, it punctured the cell, leading to sudden and widespread disruption of cell contents and, often, to cell death. In most cases snapshots, i.e. maximal projections of the images collected in each z-series, were generated for analysis of the data. In the longer sequences, there were sufficient snapshot images to compile them into a movie using Premier (Adobe). In some cases, single optical sections in the same focal plane were collected continuously to monitor rapid dynamics of GFP-tagged organelles.

**Figure 2:**
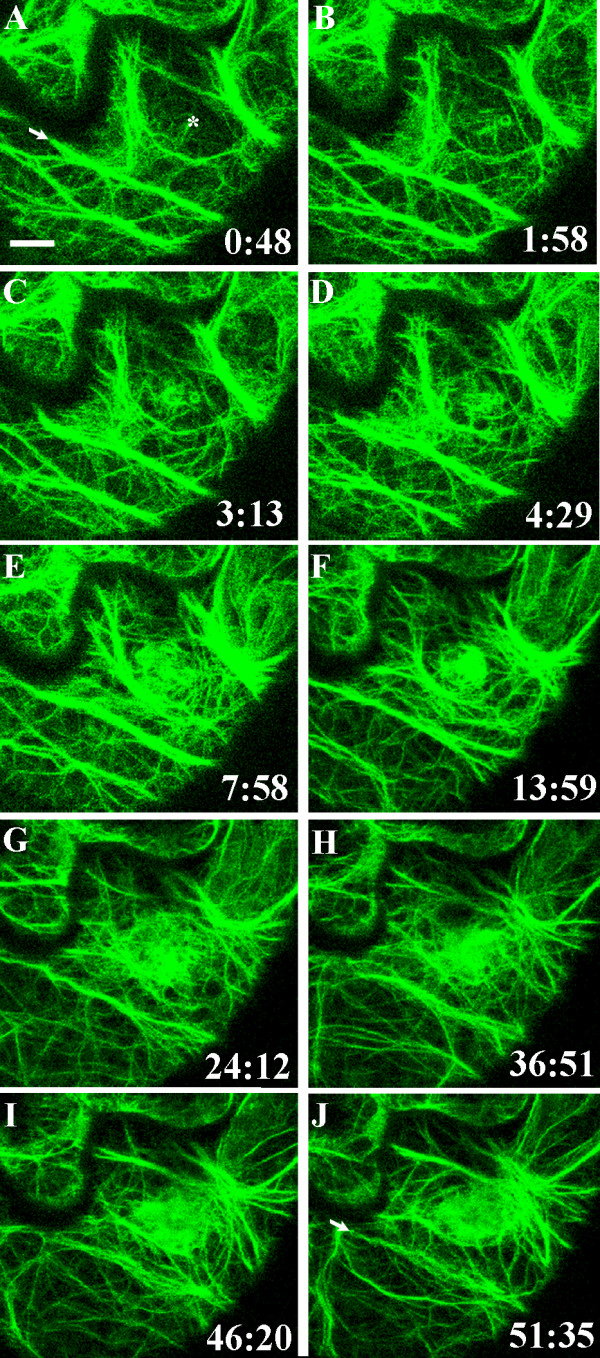
**Formation of an actin patch beneath the contact site**. Actin microfilaments visualized in the cortical cytoplasm underlying the outer epidermal cell wall in a cotyledon of *A. thaliana *expressing hTalin-GFP. The surface of the epidermal cell was touched with a glass microneedle at time 0:00 at the site indicated by the asterisk in A. Images A-J are projections of six optical sections taken at the times indicated in minutes and seconds. Actin microfilaments began to concentrate beneath the needle contact site 1 minute 58 seconds after touching the epidermal cell surface. The patch of actin continued to enlarge over the ensuing hour. The arrows in A and J indicate a thick actin cable that remains in the same position throughout the 51-minute sequence. A movie composed of images from 94 time points taken during this time is shown in Additional File 1. Bar = 10 μm.

**Figure 3:**
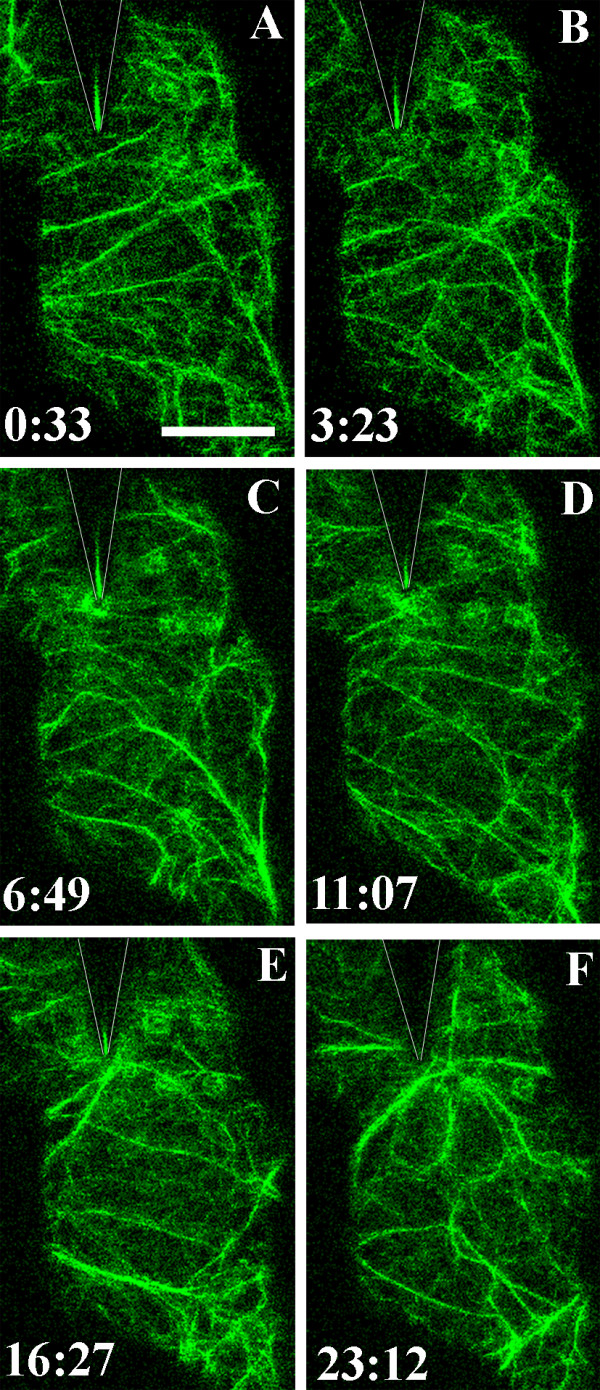
**Focusing of actin cables at the contact site**. Actin microfilaments visualized in the cortical cytoplasm underlying the outer epidermal cell wall in a cotyledon of *A. thaliana *expressing hTalin-GFP. The surface of the epidermal cell was touched with a tungsten microneedle at time 0:00. The position of the needle is indicated by the shadow outlined by the V-shaped lines. The position of the tip of the needle is shown by a line of reflected light in many images. Images A-F are projections of six optical sections taken at the times indicated in minutes and seconds. Actin microfilaments began to concentrate beneath the needle contact site 3 minutes 23 seconds after touching the epidermal cell surface. The density of the actin patch fluctuated over the ensuing 20 minutes. Between 11–23 minutes after contact, actin cables became focused on the contact site. A movie showing focusing of the actin cables in this experiment is shown in Additional File 3. Bar = 10 μm

**Figure 4:**
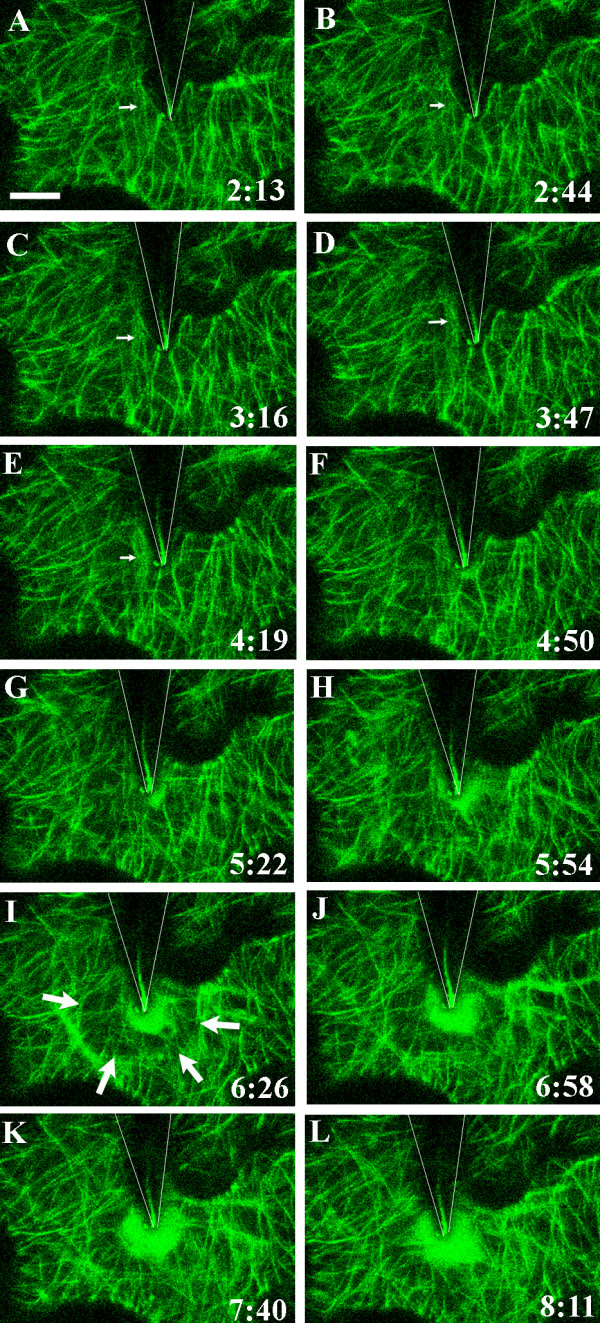
**Changes in the microtubule network beneath the point of contact**. Microtubules visualized in the cortical cytoplasm underlying the outer epidermal cell wall in a cotyledon of *A. thaliana *expressing TUA6-GFP [70]. The surface of the epidermal cell was touched with a tungsten microneedle at time 0:00. The position of the needle is indicated by the shadow outlined by the V-shaped lines. The position of the tip of the needle is shown by a line of reflected light. Images A-L are projections of five optical sections taken at the times indicated in minutes and seconds. The first sign of a reaction in the array occurs at 3 minutes 16 seconds when a linear region of diffuse fluorescence appears to the left of the needle tip (small arrow in C). A more intense cloud of diffuse fluorescence subsequently forms at the needle tip (F-L). At the same time, a microtubule-depleted zone about 20 μm in diameter forms around the needle tip as indicated by the arrows in I. A movie showing the dynamics of microtubule rearrangement in this experiment is shown in Additional File 5. Bar = 10 μm.

**Figure 5:**
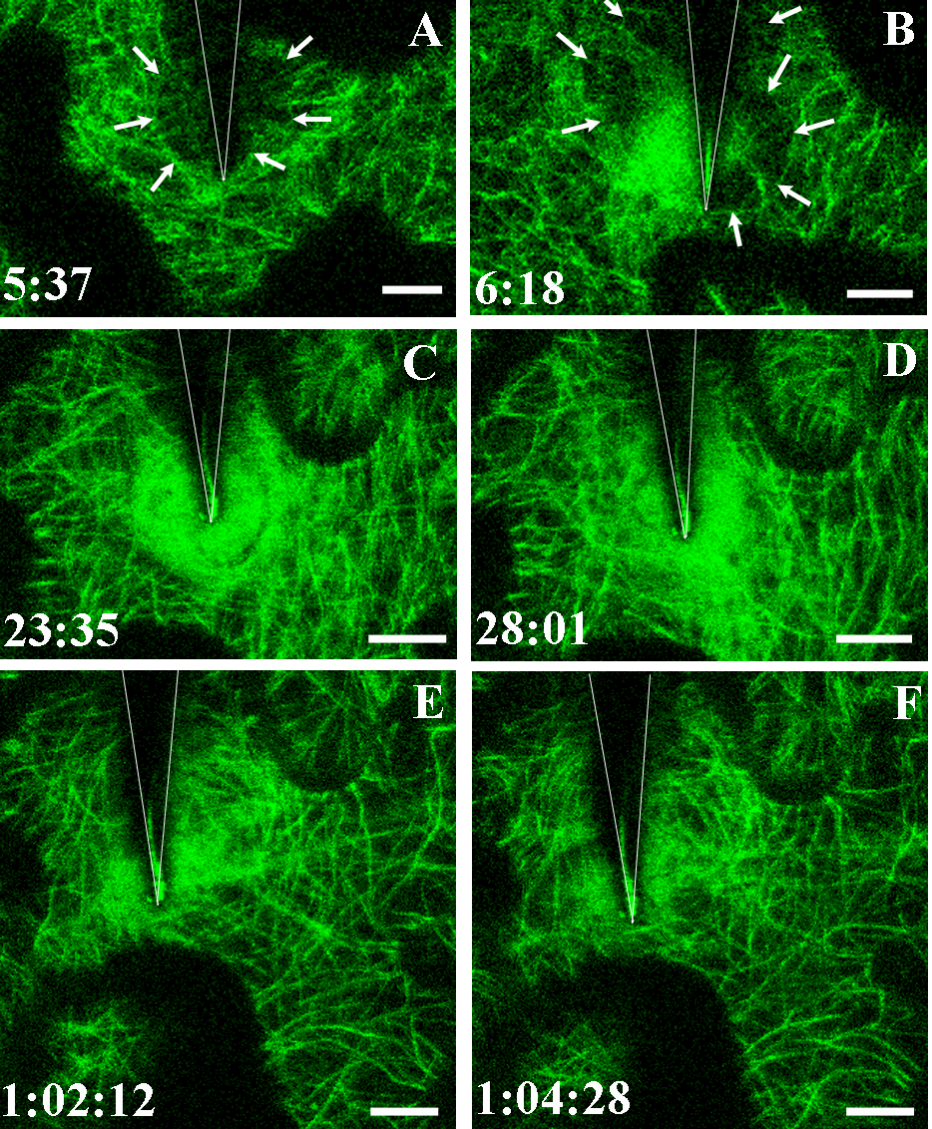
**Alterations in microtubule arrays underlying the point of contact**. Images in A and B are from two different experiments to that shown in Figure 4 and illustrate the localized microtubule depolymerization and formation of the microtubule-depleted zone (arrows) beneath the point of contact with the needle (outlined by the V-shaped lines). C-F are selected images taken at later time points in the same experiment shown in Figure 4. In C and D, taken 23–28 minutes after contact, the diffuse cloud of fluorescence fills the region previously occupied by the microtubule-depleted zone. In E and F, taken 1 hour after contact, the cloud of diffuse fluorescence has diminished and a subset of microtubules forms a circumferential array around the needle tip. Bar = 10 μm.

No differences in cell behaviour in response to being touched by either glass or tungsten microneedles were evident. In most cases, GFP-tagged components could be seen through the glass needle, although in some cases fluorescence intensity behind the needle was reduced (e.g. Figs. 2F–J). On the other hand, the tungsten needle blocked view of organelles behind it and the position of the tip of the needle could be visualized by a line of reflected light (e.g. Figs. 3, 4, 5).

### Aggregation of actin microfilaments underneath the contact site

Before being touched by the microneedle, actin microfilaments in the cortical cytoplasm underlying the outer epidermal cell wall in the *A. thaliana *cotyledons formed a lattice of thick and thin cables surrounding a dispersed network of fine filaments (Fig. 1A). Touching the surface of the cotyledon with the microneedle led to rapid changes in the arrangement of actin filaments within the cell. The first changes were observed about 3 minutes after needle contact (range = 118–246 s; mean = 177 ± 51 s, n = 6) and consisted of the appearance of a patch of fine filaments at the contact site (Fig. 2). The meshwork of actin filaments quickly assembled into a roughly circular, brightly fluorescent patch about 10 μm in diameter. The arrangement of the filaments and the morphology of the patch changed between consecutive images taken 20–30 s apart, indicating movement, polymerization and/or depolymerization of the actin microfilaments in the array. Actin in other regions of the cell was generally more stable, with actin cables in particular showing only minor alterations over 10–20 minute periods (Fig. 2; Additional Files 1, 2). The position of the thick actin cable indicated by the arrow in Fig. 2, for example, was maintained throughout the 51-minute observation period. If the microneedle were lifted off the cell surface, the actin patch dispersed (Additional File 2). When the microneedle was again brought into contact with the epidermal cell surface, the patch reformed within 4 minutes (Additional File 2).

In three of four sequences that documented changes during needle contact for more than 15 minutes, actin cables become focused on the point of contact 15–20 minutes after the surface was touched (Fig. 3; Additional File 3). In one sequence, the fluorescence beneath the needle tip was extremely bright and individual microfilaments could not be distinguished within it (Additional File 4). The shape and position of this cloud of bright diffuse fluorescence changed continuously during the 27 minutes of observation.

### Changes to the cortical microtubule arrays beneath the contact site

Before touching the surface of the cotyledon, the organization of microtubules in the cortical cytoplasm of the epidermal pavement cells differed depending on the region of the cell. In the neck region between lobed extensions, microtubules formed parallel arrays aligned transversely to the direction of extension (Fig. 1B). Within the lobes, the cortical microtubules were less well organized and displayed no predominant orientation.

The first changes in the cortical microtubule arrays were detected about 3 minutes (range = 129 – 259 s; mean = 196 ± 56 s; n = 4) after contact of the microneedle with the outer epidermal cell wall. Three main responses were observed within the microtubule arrays. (1) Linear regions of diffuse fluorescence appeared, often where a microtubule strand had previously occurred (Fig. 4A–E). (2) A dense cloud of bright fluorescence appeared close to the needle tip (Fig. 4F–L; Additional File 5). (3) A ring of cytoplasm containing a reduced density of microtubules developed around the fluorescent cloud at the needle tip, thus forming a microtubule-depleted zone about 20 μm in diameter around the contact site (Figs. 4, 5A, 5B; Additional File 5). Formation of this microtubule-depleted zone accompanied early development of the cloud of diffuse fluorescence until about 10 minutes after needle contact. With continued expansion of the cloud of diffuse fluorescence, the microtubule-depleted zone was no longer discernible (Figs. 5C–F; Additional File 5).

When the snapshots (maximum projections) of each z-series of optical sections are compiled into a movie, the dynamics of the alterations within the microtubule arrays surrounding the contact site are evident (Additional Files 5, 6). The three responses described above are seen to occur within a framework of microtubule strands that are relatively stable, undergoing only minor changes during much of the hour of observation. The size and shape of the cloud of bright fluorescence at the needle tip changes between consecutive z-series taken at intervals of about 30 s and the cloud appears as a swirling region of locally concentrated GFP-tubulin. If the needle moved across the cotyledon surface, the dense cloud of diffuse fluorescence moved with it to remain beneath the contact site (Additional Files 5, 6). The linear regions of diffuse fluorescence fluctuate rapidly. Some appear to radiate out from the central fluorescent cloud, others come and go, often appearing to move laterally before disappearing. Analysis of individual optical sections indicates that the linear diffuse fluorescent strands lie below the cortical array, i.e. they are further from the plasma membrane than the more stable cortical array. In the experiment illustrated in Figs. 4, 5C–F and Additional File 5, about 1 hour after touching the cotyledon surface with the needle, the density of the cloud of diffuse fluorescence diminished and the cortical array of microtubules could be seen to form a roughly circumferential arrangement surrounding the point of contact with the needle (Figs. 5E, 5F; Additional File 5).

Observations of microtubule arrays in control *A. thaliana *cotyledons that had not been touched with a microneedle revealed that neither the dense cloud of fluorescence nor the circumferential arrangement of cortical microtubules was seen in these cells. However, mobile linear regions of diffuse fluorescence were present at different locations from time to time (Additional File 7). As in the cells that had been touched with the needle, the diffuse strands lay beneath the stable cortical array of well-defined microtubules.

### Reorganization of the ER beneath the contact site

**Figure 6:**
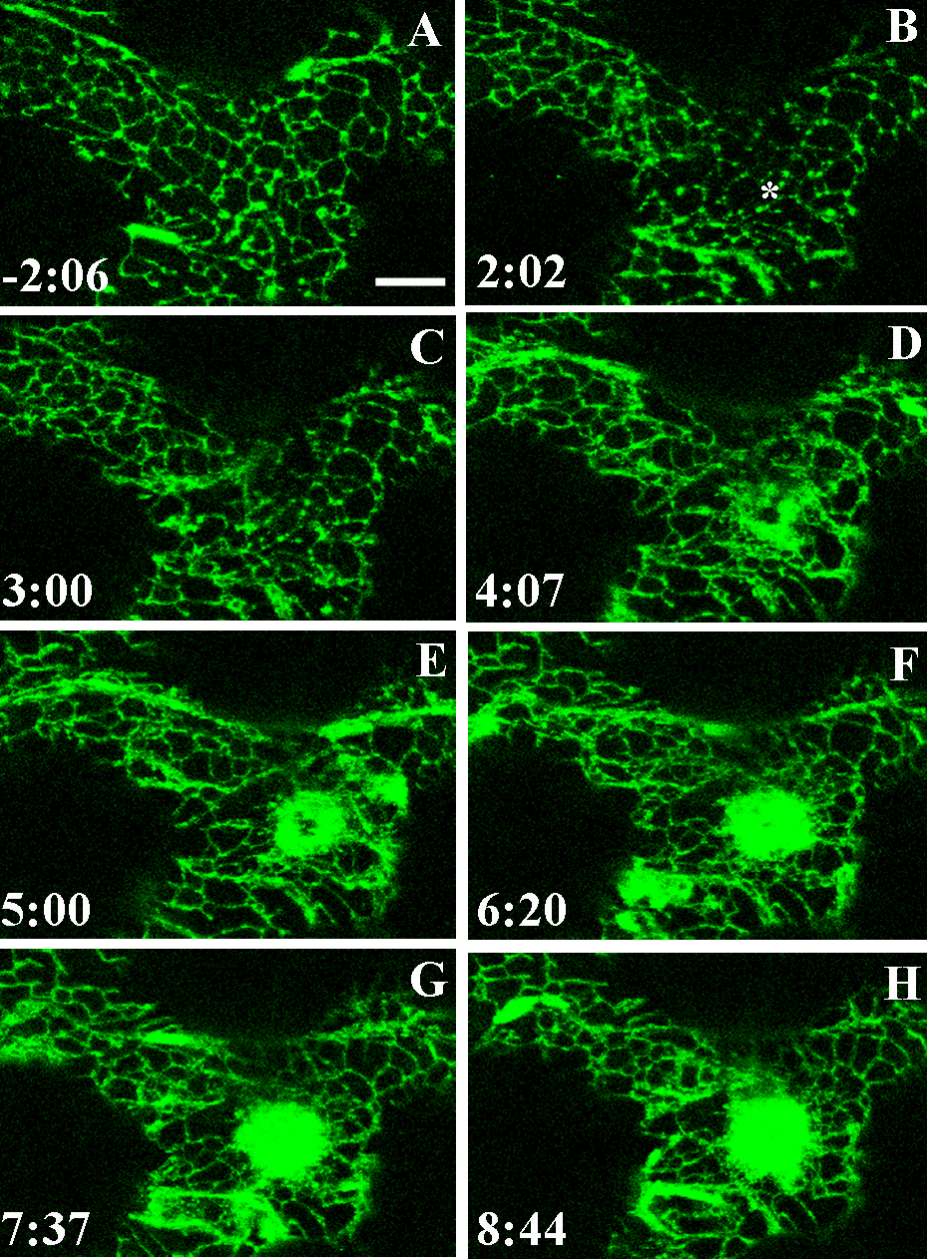
**Accumulation of ER at the point of contact**. ER visualized in the cortical cytoplasm underlying the outer epidermal cell wall in a cotyledon of *A. thaliana *expressing GFP-KKXX [71]. The surface of the epidermal cell was touched with a glass microneedle at time 0:00 at the position marked by the asterisk in B. Images A-F are projections of five optical sections taken at the times indicated, with the image in A being collected about 2 minutes before the cotyledon was touched with the needle. ER began to aggregate beneath the tip of the needle 3–4 minutes after touching the epidermal cell surface. Strands of diffuse fluorescence form near the contact sites and a cloud of bright fluorescence forms and expands. Bar = 10 μm

Before touching the epidermal cells with the needle, the ER formed a lace-like network in the cortical cytoplasm (Figs. 1C, 6A; Additional File 8). After bringing the needle into contact with the outer epidermal cell wall, there was often a rapid, sometimes transient response in which the ER lattice in the vicinity of the needle took on a beaded appearance as the fluorescence of the tubular sections of ER diminished and the interstices of the lattice became more prominent (Fig. 6B). More extensive changes in ER distribution were detected within about 4 minutes (range = 146–289 s; mean =227 ± 73 s; n = 3) of touching the cotyledon surface. At this time, strands of diffuse fluorescence formed near the contact site although they were not necessarily focused on the site of needle contact (Figs. 6D–H). At the same time, a circular zone of bright diffuse fluorescence accumulated at the contact site. The size and intensity of this brightly fluorescent region increased with time (Figs. 6D–H; Additional File 9). If the needle were lifted from the cotyledon surface, the brightly fluorescent aggregation dispersed; if the needle were moved across the cotyledon surface, the fluorescent aggregation moved to remain beneath the contact zone (data not shown).

In order to determine if all the changes observed after touching the cotyledon with the needle, as described above, were directly associated with a touch response, the structure of the ER was also observed in cotyledons that were not touched with the microneedle (Additional File 8). Imaging of the ER network in these control, untouched cells revealed that while the transient beading and formation of the circular fluorescent zone were not observed, strands of diffuse fluorescence similar to those seen in the cells that had been touched sometimes formed (Additional File 8, images A-C, E and F). These strands of diffuse fluorescence in the control cells were formed less frequently and were more transient than those seen in the cells that were touched with the needle.

### Clustering of peroxisomes beneath the site of contact

**Figure 7:**
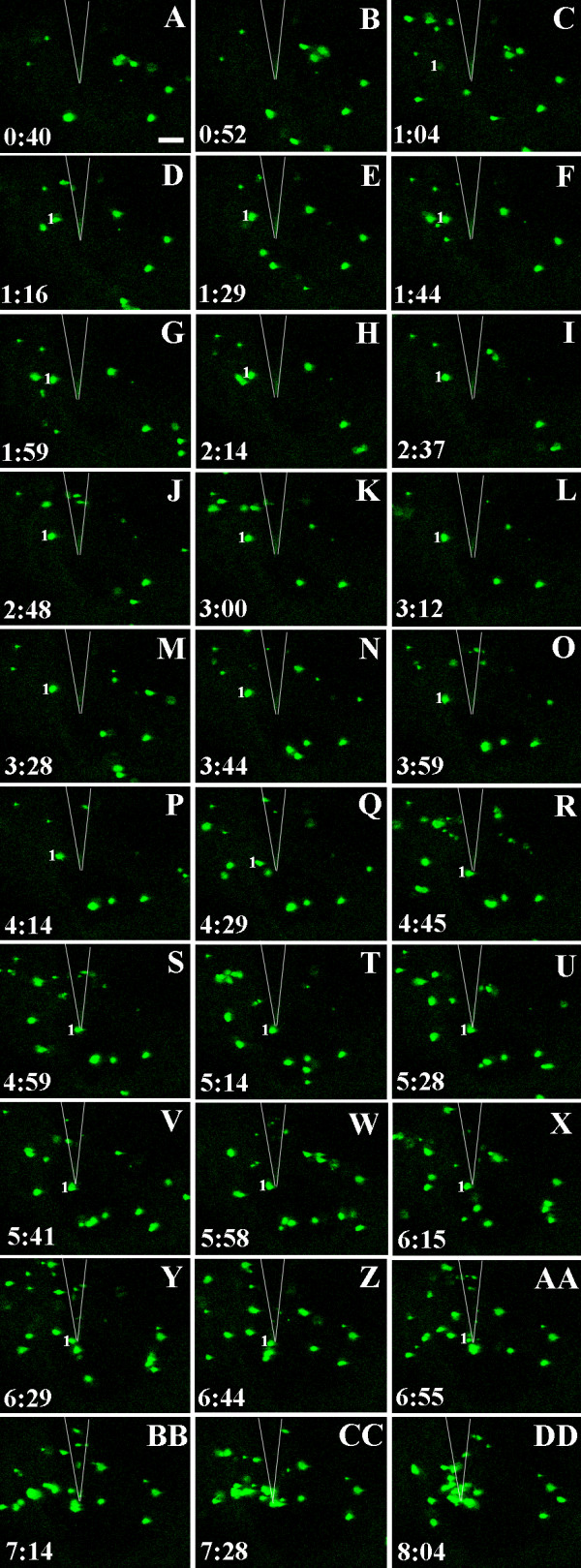
**Clustering of peroxisomes at the point of contact**. Peroxisomes visualized in the cortical cytoplasm underlying the outer epidermal cell wall in a cotyledon of *A. thaliana *expressing PTS-GFP [46]. The surface of the epidermal cell was touched with a tungsten microneedle at time 0:00. The position of the needle is indicated by the white V-shaped lines at the top of the image. Images A-DD are projections of three optical sections taken at the times indicated in minutes and seconds. The peroxisome labeled "1" becomes immobilized about 10 μm to the left of the needle tip 1 minute after touching the cotyledon surface. About 3 minutes later, this peroxisome moves to the very tip of the needle. Other peroxisomes accumulate in the vicinity of the needle tip before becoming clustered there after about 7 minutes. A movie showing peroxisome aggregation in this experiment is shown in Additional File 10. Bar = 10 μm.

Before touching the surface of the epidermal cell with the microneedle, peroxisomes were distributed throughout the cytoplasm and most were highly mobile, moving rapidly across the field of view (Fig. 1D; Additional File 10). About 5 minutes after touching the surface of the cotyledon with the needle (range = 266–324 s; mean =297 ± 29 s; n = 3), peroxisomes began to accumulate beneath the needle tip (Fig. 7Q; Additional File 10). In general, once a peroxisome had moved into the cluster at the needle tip, apart from a small jiggling motion, it remained there throughout the remainder of the observation period. Even after a cluster of peroxisomes had formed beneath the needle, other peroxisomes that were more than about 20–30 μm away from the point of contact continued to move around the cell. With time, however, the local area became depleted of peroxisomes that were not associated with the cluster at the needle tip.

## Discussion

Plant cells are able to respond rapidly to potential fungal or oomycete pathogens on their surface. As the pathogen infection structures develop and attempt to penetrate the epidermis, basal resistance mechanisms are mobilized and in most cases succeed in inhibiting the establishment of disease by non-adapted pathogens. Within the plant cell in contact with the pathogen, cytoplasmic streaming accelerates, cytoplasmic strands become focused on the infection site and a cytoplasmic aggregate forms under the pathogen cell [1,13,25]. Immunofluorescence labelling, GFP-tagging of cell components and pharmacological studies have shown that these changes in the distribution and behaviour of the cytoplasm are associated with and dependent upon reorganization of the actin cytoskeleton which becomes focused on the infection site [6,20,21,26-30]. Reorganization of the actin cytoskeleton is also accompanied by rearrangement of ER and aggregation of dictyosomes and peroxisomes at the infection site [20,31]. These changes are believed to facilitate localized secretion of cell wall material and antimicrobial compounds at the infection site [15,16]. In addition to normal wall constituents, the thickened regions of cell wall, referred to as wall appositions, contain callose, phenolics, phytoalexins and H_2_O_2 _that together strengthen the wall to make it a more effective barrier against pathogen ingress and provide localized concentrations of toxins that inhibit and kill the invading pathogen [11,13,16,32]. Basal resistance is induced in response to non-adapted, avirulent and virulent fungal and oomycete pathogens alike [20], suggesting the existence of a triggering factor common to all these potential pathogens that can be detected by the plant. One such trigger could be the pressure exerted by the pathogen as it attempts to penetrate the plant surface.

Plants can respond rapidly to mechanical stimulation. Within 30 minutes, touching a plant induces wide-ranging changes in gene expression, including the up-regulation of disease resistance genes [33-35]. It also leads to rapid changes in intracellular organization. Touching the cell surface with a glass or tungsten microneedle or capillary can cause chloroplast movement away from, or nuclear and cytoplasmic migration towards, the contact site [36-38]. Removal of the stimulus results in disappearance of the cytoplasmic aggregation and a return of the nucleus to its former position [37,38]. The present study of *Arabidopsis *plants containing GFP-tagged cell components demonstrates that touching the plant surface also induces a reorganization of subcellular components similar to that observed during attempted infection. Within 3 to 5 minutes of touching the cotyledon surface, actin, ER and GFP-tubulin begin to form dense patches under the contact site and peroxisomes begin to cluster in the cytoplasm beneath the needle tip. The subcellular responses are highly dynamic, with the morphology of the patches of actin, ER and GFP-tubulin changing continuously, including tracking the needle tip when it moves across the surface and dispersing within minutes of removal of the needle's pressure.

Studies of actin arrays in transgenic plants expressing a number of different constructs encoding GFP-tagged actin binding domains have indicated that in some cases not all components of the actin cytoskeleton are visualized and/or that the dynamics and organization of the actin array may be disturbed [39-44]. There is, however, no evidence to indicate that these problems occur in the GFP-hTalin plants used in the present study. Indeed the rapidity with which the actin array becomes focused on the contact site, the similar rapidity with which it disperses on removal of the pressure and the fact that this response time is similar to that of the microtubule array, all suggest that actin dynamics are not hampered in this line of GFP-hTalin plants. In the experiment that generated the GFP-hTalin plants, individual lines exhibited a range of GFP fluorescence intensities [20]. The line chosen for subsequent use was selected for having moderate levels of transgene expression, as indicated by the levels of GFP fluorescence, and no phenotypic abnormalities [20]. In addition to the absence of any indication of disturbance of the actin arrays in these plants in our experiments, this *Arabidopsis *GFP-hTalin line has also been used in investigations of the role of actin in regulating the dynamics of plastid stromule morphology and behaviour [45]. Stromule formation is a highly delicate process that is easily perturbed, however, no differences in the morphology or movement of the plastids or stromules between wild-type and the GFP-hTalin plants were found. Together, these results give evidence of the integrity and normal dynamics of the actin cytoskeleton in the transgenic plants used in the current study.

With time, the initial concentrations of actin, ER and GFP-tubulin continue to consolidate. Some existing actin cables in the region surrounding the contact site are dismantled before new, or reoriented, cables become focused on the contact site. Much accumulated evidence indicates that the radial actin array is responsible for delivery of various cell components, including peroxisomes, dictyosomes and secretory vesicles, to the site of attack [15,17,46-48]. In addition to achieving localized secretion of cell wall materials and toxins, proximity of peroxisomes to the site of infection is thought to facilitate their anti-microbial functions [49-51].

Continued response to touch included the development of a microtubule-depleted zone around the contact site. Similar localized regions of microtubule depolymerization have been reported during infection of parsley, soybean and barley by *Phytophthora infestans, P. sojae and Erysiphe graminis *f. sp.*hordei*, respectively [52-54]. Widespread microtubule depolymerization has also been reported to occur several hours after treatment of tobacco and *Arabidopsis *suspension-cultured cells with cryptogein elicitin and *Verticillium *toxin, respectively [55-57]. Microtubule depolymerization is likely to increase the level of tubulin monomers, dimers or oligomers in the cytoplasm, giving rise to the dense cloud of diffuse fluorescence seen below the contact site (e.g. Figs. 4H–L, 5B–E). The dynamic behaviour of the GFP-tubulin and ER clouds is likely to arise through continued actin-driven cytoplasmic motility, as indicated by peroxisome movement throughout much of the observation periods. In our study, disturbance of the ER network and microtubule cytoskeleton through mechanical stimulation also led to the appearance of diffuse strands of ER or microtubules that moved within the cortical cytoplasm. Similar strands were seen in control cells that had not been touched with the microneedle but they were much less frequent and were not confined to any particular location in the cell. The diffuse strands observed in the GFP-tubulin-expressing plants were similar to the blurry microtubules described by Cyr and colleagues in their studies of the dynamics of cortical microtubule arrays in tobacco cells expressing MDB-DsRed or YFP-TUA6 [58]. These authors propose that the blurry images of microtubules are due to movement of microtubules that are not anchored to the plasma membrane. The diffuse microtubule strands observed in the present study were at a greater distance from the plasma membrane than the array of sharply-focused microtubules and are thus unlikely to be attached to the plasma membrane. In our studies of microtubule arrays in cells responding to mechanical stimulation, in most cases these diffuse microtubule strands subsequently disappeared. One interpretation of these data is that, having detached from the plasma membrane, the microtubules or microtubule bundles are less stable and depolymerize. This phenomenon may be part of the normal dynamics of microtubule arrays but may also be an important mechanism for remodeling microtubule arrays following mechanical stimulation by biotic or abiotic factors.

One might question whether or not a fungal hypha or other infection structure is capable of exerting a pressure or force sufficiently large to be perceived by the plant cell. In fact, there is little doubt that it can. A variety of technical approaches have now been used to calculate or measure the force exerted by fungal and oomycete hyphae and appressorial penetration pegs and the values obtained typically lie within the range of 5–100 μN [59-63]. Given the diminutive size of the structures involved, it is difficult to fully appreciate the magnitude of these forces but to put them in perspective, if we could exert the same force per unit area with a finger, we could hold a 25–500 kg weight against gravity!

The force exerted by an invading pathogen will depend on the pathogen cell's turgor pressure and the area and properties of its cell wall in contact with the underlying plant cell. Turgor pressure provides the basis of the invasive force although the actual pressure applied to the host cell will be decreased by the resistance of the pathogen wall to extension [64]. The force (in μN) is equal to the pressure (in MPa, i.e. μN μm^-2^) multiplied by the contact area (in μm^2^). Thus, if the wall exerts minimal yield resistance, a hypha with a cross-sectional area of 300 μm^2 ^(about 20 μm in diameter) and a turgor of 0.4 MPa would exert a force of 120 μN [59]. In most cases, the forces that have been measured are less than those maximally possible for a given turgor pressure and it appears, at least for hyphae, that the mechanical strength of the wall results in only about 10% of the available force actually being applied by the fungal hypha [59]. However, this may not be the case for appressorial penetration pegs, as discussed below.

Fungal and oomycete hyphae typically generate turgor pressures of 0.2–0.7 MPa [59]. With a cross-sectional area of 80–500 μm^2^, these hyphae have a contact area of up to 500 times that of the penetration peg produced by appressoria of *Colletotrichum graminicola *or *Magnaporthe grisea*. Thus, in order to generate an invasive force within the range cited above, these fungal appressoria accumulate high concentrations of glycerol that generate turgor pressures of the order of 6–8 MPa, a pressure 30–40 times greater than an average car tire [64-66]. Evidence suggests that the yield threshold of the penetration peg is minimal such that essentially all the turgor pressure is applied in the generation of the invasive force by the penetration peg [62,64]. Thus, the localized forces exerted by invading fungi and oomycetes are substantial and, like the mechanical stimulation used in the study, may be detected by the plant cell and used to trigger basal defence.

A number of studies have shown that cytoplasmic aggregation and actin reorganization can occur in the plant cell below hyphae or beneath appressoria before a penetration peg is discernible, suggesting that, at least in some cases, the plant cell can detect the presence of hyphae or appressoria on their surface before invasion begins [1,7,13,21,25]. However, given the difficulties of knowing when the pathogen has strengthened its attachment to the plant surface sufficiently to maintain contact during invasion, when an appressorium has gained full turgor or when penetration is first initiated, it is difficult to assess how quickly the plant can respond to the presence of the pathogen. In addition to demonstrating that touching the cotyledon surface with a microneedle induces similar subcellular reorganization to that observed during pathogen infection, this form of mechanical stimulation allows a more precise determination of the dynamics of the response than is possible during plant-pathogen interactions. Thus, while cytoplasmic aggregation, actin rearrangement or microtubule depolymerization have been observed to occur 15 minutes after inoculation or 20–30 minutes before a penetration peg becomes visible [1,25], the studies reported here indicate that these subcellular responses can occur within 3–4 minutes of stimulation. Touch can induce a transient increase in cytoplasmic calcium in a similar or even shorter timeframe [67,68]. As proposed for the mechano-response of chloroplasts [69], these observations are consistent with stretch-activated channels in the plant plasma membrane having a role in recognition of the pathogen's presence and induction of a signal transduction cascade involving transient calcium elevation that quickly results in the subcellular reorganization employed during the basal plant defence response.

## Conclusion

Our study shows that mechanical stimulation applied by gently touching the surface of a plant cell with a glass or tungsten needle induces the same subcellular reorganization observed following inoculation of a wide range of plant species with non-adapted, avirulent and virulent fungal or oomycete pathogens. Actin microfilaments and cables become focused on the site of contact, and ER and peroxisomes accumulate beneath the needle tip. A sub-population of the cortical microtubules surrounding the point of contact depolymerizes, generating a microtubule-depleted zone around a patch of concentrated GFP-tubulin subunits. Reorganization of all four cell components begins within 3 to 5 minutes of touching the cell surface with the needle, demonstrating the rapidity with which the plant cell can respond to mechanical stimulation. Our study provides strong evidence indicating that plant cells can detect the force exerted by fungal or oomycete cells as they attempt to invade the plant epidermis, thus triggering the basal defence response that culminates in site-directed secretion and the development of wall appositions which successfully inhibit ingress of most potential pathogens.

## Methods

### Plants

Transgenic *A. thaliana *(Columbia ecotype) expressing GFP-tagged components were as follows: GFP-TUA6 (α-tubulin), kindly supplied by Dr T. Hashimoto [70]; GFP-PTS1 (peroxisome targeting sequence), kindly supplied by Dr S. Mano [46]; GFP-tm-KKXX (GFP with Cf-9 transmembrane domain and cytosolic tail with C-terminal dilysine motif that confers ER localization) [71]; and GFP-hTalin [20]. Seeds were surface sterilized and grown at 25°C (16 h light per day) on nutrient agar plates as described previously [20].

### Microscopy

*Arabidopsis *cotyledons expressing GFP-tagged components were placed adaxial side up on a smear of vaseline on a microscope slide and held in place by partially covering the cotyledon with a coverslip. The exposed portion of the cotyledon was viewed using a 63×, NA 0.9 dipping objective on a TCS SP2 confocal microscope (Leica, Germany). GFP fluorescence was excited by an argon-ion laser at 488 nm and emission of GFP-tagged components in the epidermal cells captured between 495 and 520 nm. Images shown in the majority of figures are maximal projections of z-series of optical sections through the cortical cytoplasm underlying the outer epidermal cell wall. The images were generated using Leica Lite software, stored as TIF files and processed with Adobe Photoshop 7.0 software (Adobe Systems Inc., USA).

Gentle mechanical pressure was applied to the outer epidermal cell wall by bringing a fine glass (1.0 mm diameter glass capillaries, 1–5 μm diameter at tip) or tungsten (0.25 mm diameter tungsten dissecting probes, World Precision Instruments Inc., USA, 1–2 μm diameter at tip) microneedle into contact with the cotyledon surface using a hydraulic micromanipulator (Narashige, Japan). The distance of the tip of the needle from the cotyledon surface was determined by comparing the focal plane of GFP-tagged components in the cortical cytoplasm with that of the needle tip.

## Authors' contributions

ARH conceived of the study, participated in its design and execution, and drafted the manuscript; DT contributed to the conception and planning of the study; RW participated in design of the study and performed the micromanipulation and microscopy. All authors read and approved the final manuscript.

## Supplementary Material

Additional file 1 **Rapid development of a patch of actin microfilaments at the contact site**. Movie composed of 94 images of the actin array underneath the point of contact with a glass microneedle in *A. thaliana *expressing hTalin-GFP. Each image in the movie is a projection of six optical sections through the cortical cytoplasm underlying the outer epidermal cell wall. The movie commences at the time of touching the cell surface with the needle and ends 1 hour and 5 minutes later. The time between most images is about 40 seconds. Selected images from the movie are illustrated in Fig. 1. The movie plays at about 200 times real-time. Click here for file

Additional file 2 **Formation, dispersal and re-formation of an actin patch at the contact site**. Actin microfilaments visualized in the cortical cytoplasm underlying the outer epidermal cell wall in a cotyledon of *A. thaliana *expressing hTalin-GFP. The surface of the epidermal cell was touched with a glass microneedle at the site indicated by the asterisk in A. Times in minutes and seconds show elapsed time after the image in A. Images A-C show formation of a patch of actin microfilaments about 6 minutes after touching the cell surface. Needle contact was made about 10 minutes after the image in A was taken. Images D-F show dispersal of the actin patch after the needle lifted off the cotyledon. Images G-I show reformation of the actin patch when the needle was again brought into contact with the cell at the same location. The image in G was taken 1 minute after re-positioning the needle to touch the surface again. Images are projections of 7 (B, G-I), 8 (C, E), 9 (D), 12 (F) or 13 (A) optical sections. Bar = 10 μm. Click here for file

Additional file 3 **Focusing of actin cables on the contact site**. Movie composed of 53 images of the actin array underneath the point of contact with a tungsten microneedle in *A. thaliana *expressing hTalin-GFP. Each image in the movie is a projection of six optical sections through the cortical cytoplasm underlying the outer epidermal cell wall. The position of the needle tip is indicated by the vertical line of reflected light. The movie commences just before touching the cell surface with the needle and ends 28 min later. The time between most images is 20–25 s. During the second half of the movie sequence, actin cables become focused on the contact site. The movie plays at about 150 times real-time. Selected images from the sequence are illustrated in Fig. 2. Click here for file

Additional file 4 **Dynamics of a bright cloud of actin at the point of contact**. Movie composed of 73 images of the actin array underneath the point of contact with a tungsten microneedle in *A. thaliana *expressing hTalin-GFP. Each image in the movie is a projection of five optical sections through the cortical cytoplasm underlying the outer epidermal cell wall. The position of the needle is indicated by the V-shaped shadow. The movie commences just before touching the cell surface with the needle and ends 28 minutes later. The time between most images is about 20 seconds. Accumulation of actin below the point of contact forms a bright patch of fluorescence that appears to swirl around the needle tip. The movie plays at about 150 times real-time. Click here for file

Additional file 5 **Changes in the microtubule array beneath the point of contact**. Movie composed of 115 images of the microtubule array underneath the point of contact with a tungsten needle in *A. thaliana *expressing TUA6-GFP [70]. Each image in the movie is a projection of five optical sections through the cortical cytoplasm underlying the outer epidermal cell wall. The position of the needle is indicated by the V-shaped shadow (top centre). The movie commences just before touching the cell surface with the needle and ends 1 hour 17 minutes later. The time between most images is about 30–40 seconds. A bright diffuse cloud of GFP-tubulin begins to accumulate at the point of contact about 4 min after touching the cell with the needle. Linear arrays of diffuse fluorescence appear to sweep below the cortical microtubule array. The movie plays at about 200 times real-time. Click here for file

Additional file 6 **Dynamic changes in the microtubule array as the contact point moves across the cell wall**. Movie composed of 95 images of the microtubule array underneath the point of contact with a tungsten needle in *A. thaliana *expressing TUA6-GFP. Each image in the movie is a projection of five optical sections through the cortical cytoplasm underlying the outer epidermal cell wall. The position of the needle is indicated by the V-shaped shadow (top centre). The movie commences just before touching the cell surface with the needle and ends 1 hour 11 minutes later. The time between most images is about 30–40 seconds. A bright diffuse cloud of GFP-tubulin begins to accumulate at the point of contact about 3.5 minutes after touching the cell with the needle. As the needle drifts across the cotyledon surface, the cloud of concentrated GFP-tubulin moves with it to remain beneath the point of contact. When the needle tip moves from one cell to an adjacent cell the fluorescent cloud dissipates in the first cell and forms in the second. The movie plays at about 200 times real-time. Click here for file

Additional file 7 **Dynamics of the microtubule array in a control cotyledon**. Movie composed of 100 images of the microtubule array underneath the point of contact with a tungsten needle in *A. thaliana *expressing TUA6-GFP. All images in the movie are single optical sections in the same focal plane taken at intervals of 3–4 seconds for a period of 6 minutes. Against a framework of stable microtubules in the cell cortex just below the cell wall, mobile, linear strands of diffuse fluorescence sweep through the cell beneath the cortical array. The movie plays at about 18 times real-time. Click here for file

Additional file 8 **Dynamics of the ER in epidermal cells in control cotyledons**. ER in the cortical cytoplasm underlying the outer epidermal cell wall in a cotyledon of *Arabidopsis thaliana *expressing GFP-KKXX [71]. The cotyledon has been mounted onto a microscope slide but had not been touched with a microprobe. In general, the network of ER is stable and its organization does not change during the observation period. However, some transient flaring of strands of diffuse fluorescence occurs in the top right hand corner of the cell in the first three images (arrowheads) and near the left hand side of the cell in the images taken at 4 minutes 18 seconds and 5 minutes 12 seconds (arrows). Bar = 10 μm. Click here for file

Additional file 9 **Dynamic changes in the organization of the cortical ER beneath the contact site**. Movie composed of 77 images of the ER in an epidermal cell touched with a tungsten needle in *A. thaliana *expressing GFP-KKXX. Each image in the movie is a projection of five optical sections; the interval between each z-series is about 35 seconds. The movie begins when the cell is touched with the needle and shows images collected over the next 52 minutes. Bright fluorescence indicative of aggregated ER accumulates beneath the contact site. The movie plays at about 200 times real-time. Click here for file

Additional file 10 **Clustering of peroxisomes at the site of contact with the microneedle**. Movie composed of 43 images of peroxisomes in an epidermal cell touched with a tungsten needle in *A. thaliana *expressing PTS-GFP [46]. Each image in the movie is a projection of three optical sections and the interval between each z-series is about 15 seconds. The movie begins when the cell is touched with the needle and shows images collected over the next 14 minutes. About 5 minutes after touching the cotyledon, peroxisomes begin to cluster beneath the tip of the needle. Selected images from the first 8 minutes of observation are shown in Fig. 6. The movie plays at about 100 times real-time. Selected images from this sequence are shown in Fig. 7. Click here for file
