# Supplementary figures and images for: Rapid and dynamic subcellular reorganization following mechanical stimulation of Arabidopsis epidermal cells mimics responses to fungal and oomycete attack
Source: BMC Plant Biol. 2008 Jun 2;8:63. doi: 10.1186/1471-2229-8-63 (PMC2435237; doi:10.1186/1471-2229-8-63)

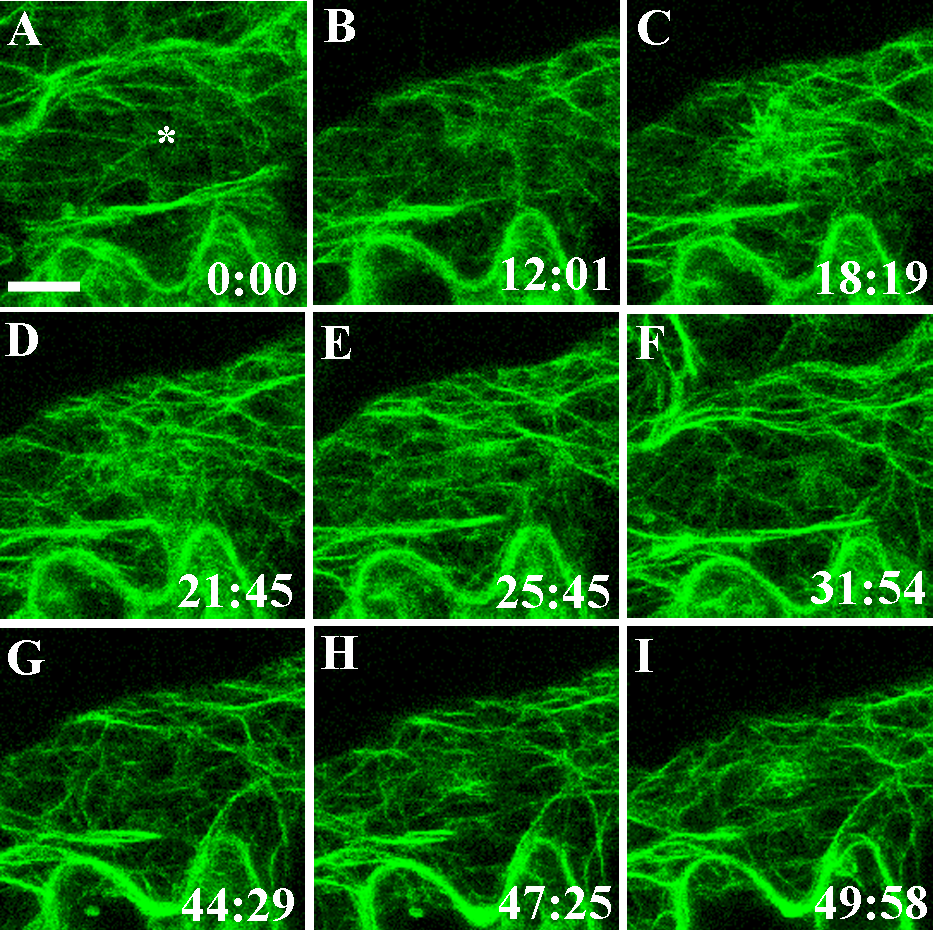

Supplement: Additional file 2 — Formation, dispersal and re-formation of an actin patch at the contact site. Actin microfilaments visualized in the cortical cytoplasm underlying the outer epidermal cell wall in a cotyledon of A. thaliana expressing hTalin-GFP. The surface of the epidermal cell was touched with a glass microneedle at the site indicated by the asterisk in A. Times in minutes and seconds show elapsed time after the image in A. Images A-C show formation of a patch of actin microfilaments about 6 minutes after touching the cell surface. Needle contact was made about 10 minutes after the image in A was taken. Images D-F show dispersal of the actin patch after the needle lifted off the cotyledon. Images G-I show reformation of the actin patch when the needle was again brought into contact with the cell at the same location. The image in G was taken 1 minute after re-positioning the needle to touch the surface again. Images are projections of 7 (B, G-I), 8 (C, E), 9 (D), 12 (F) or 13 (A) optical sections. Bar = 10 μm. [file 1471-2229-8-63-S2.tiff]

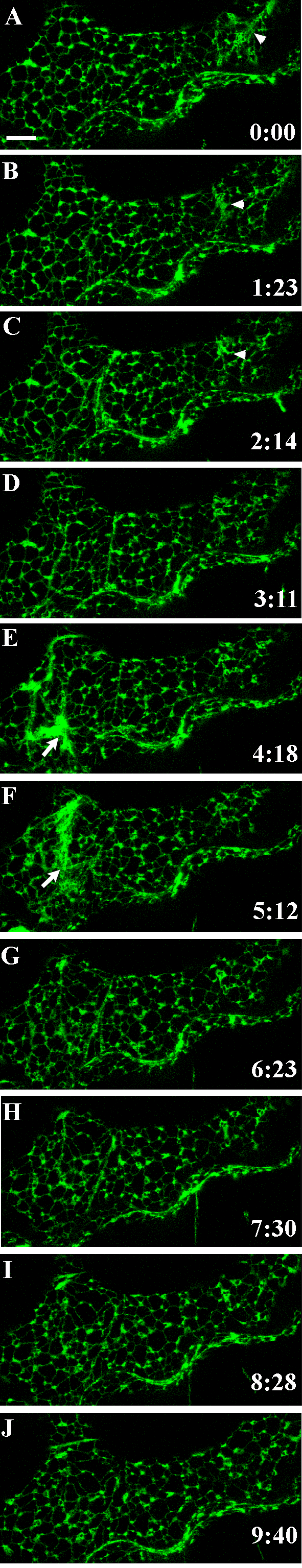

Supplement: Additional file 8 — Dynamics of the ER in epidermal cells in control cotyledons. ER in the cortical cytoplasm underlying the outer epidermal cell wall in a cotyledon of Arabidopsis thaliana expressing GFP-KKXX [71]. The cotyledon has been mounted onto a microscope slide but had not been touched with a microprobe. In general, the network of ER is stable and its organization does not change during the observation period. However, some transient flaring of strands of diffuse fluorescence occurs in the top right hand corner of the cell in the first three images (arrowheads) and near the left hand side of the cell in the images taken at 4 minutes 18 seconds and 5 minutes 12 seconds (arrows). Bar = 10 μm. [file 1471-2229-8-63-S8.tiff]
